# Pierre-Marie Bamberger Syndrome Leading to the Diagnosis and Surgical Treatment of a Localized Lung Cancer

**DOI:** 10.7759/cureus.48991

**Published:** 2023-11-18

**Authors:** Evangelos Koliakos, Dimitri Chappalley, Evangelos Kalogiannis, Sebastien Sgardello, Michel Christodoulou

**Affiliations:** 1 Division of Thoracic Surgery, Valais Romand Hospital Center, Sion, CHE; 2 Department of Visceral Surgery, CHUV (Centre Hospitalier Universitaire Vaudois) - Lausanne University Hospital, Lausanne, CHE; 3 Department of General Surgery, Hospital of Fribourg, Fribourg, CHE; 4 Department of General Surgery, Valais Romand Hospital Center, Sion, CHE

**Keywords:** non-small-cell lung carcinoma, lung neoplasms, cyclooxygenase 2, vascular endothelial growth factors, osteoarthropathy

## Abstract

Hypertrophic osteoarthropathy (HOA), manifested with digital clubbing, tubular bone periostosis, and large joint synovial effusions, exists in two forms: primary, which is the rarest form, and secondary. The latter is frequently associated with lung diseases and, in some cases, with non-small cell lung cancer (NSCLC) and is thus expressed in the form of a paraneoplastic syndrome. We report the case of a male smoker who was presented with secondary hypertrophic osteoarthropathy and was subsequently diagnosed with primary adenocarcinoma of the lung.

A 63-year-old male with a history of ischemic heart disease and heavy tobacco consumption (60 pack-years) presented with painful osteoarthritis of all four extremities. A chest computed tomography (CT), a positron emission tomography (PET) scan, and a bronchoscopy revealed a 9 cm mass within the right lower lobe without mediastinal adenopathy. Bilateral lower limb X-rays revealed osteoarthropathy of the tibia. A right lower lobectomy and mediastinal lymph node dissection were performed. Final histopathology analysis reported an advanced mixed pulmonary adenocarcinoma. The postoperative course was uneventful and the patient was discharged on postoperative day 6.

This report has highlighted the importance of clinical awareness of the association between HOA and carcinoma of the lung.

## Introduction

Hypertrophic osteoarthropathy (HOA), also known as Pierre-Marie Bamberger syndrome, was first described by Pierre Marie and Eugen von Bamberger in 1891. The syndrome presents in two forms and is characterized by hypertrophic skin and osseous tissue of the extremities presented in the form of digital clubbing, periostosis of tubular bones, and synovial effusions mainly situated in large joints. Primary HOA, also called pachydermoperiostosis, is a hereditary disorder and is less common than secondary HOA since it represents only 5% of HOA cases [1-3]. Secondary HOA is mainly associated with lung diseases such as neoplasia and chronic lung pathologies but may also be associated with a right-left shunt as part of cardiac diseases. Non-small cell lung cancer (NSCLC) is the most common pathology associated with this paraneoplastic syndrome with adenocarcinomas and epidermoid carcinomas being represented equally [3-6].

Awareness of the syndrome and its correlation with pulmonary neoplasia can help in the timely identification and treatment of pulmonary malignancies [7-9].

This is a report of a 63-year-old male smoker who presented with Pierre-Marie Bamberger syndrome and was subsequently diagnosed with a primary adenocarcinoma of the lung.

## Case presentation

**Figure 1:**
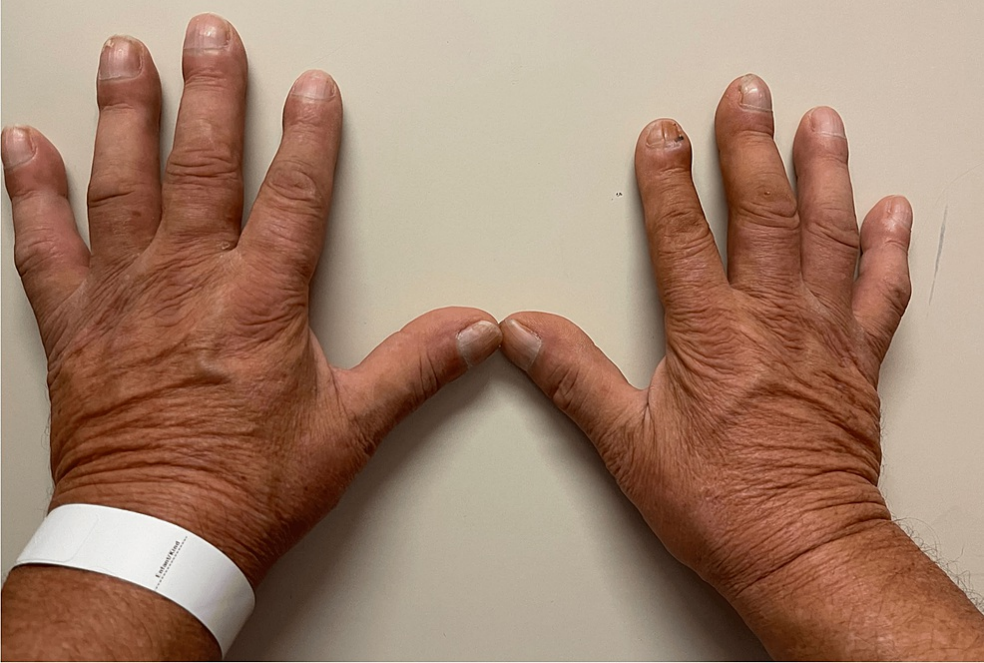
Clinical osteoarticular manifestations of Pierre-Marie Bamberger syndrome Preoperative photograph of the patient's hands demonstrating typical bilateral digital clubbing

A 63-year-old man with a history of heavy tobacco consumption (60 pack-years), ischemic heart disease, and peripheral occlusive arteriopathy consulted his physician for painful articular inflammation and edema of all four extremities for four weeks. The most prominent clinical sign was digital clubbing (Figure 1). Clinical examination also revealed synovial effusions of fingers, wrists, and ankles with a slightly decreased range of motion. The extreme motion of those articulations was painful. Further anamnesis revealed complaints of several episodes of hemoptysis during the past month, compelling his general practitioner to address the patient for chest imaging.

**Figure 2:**
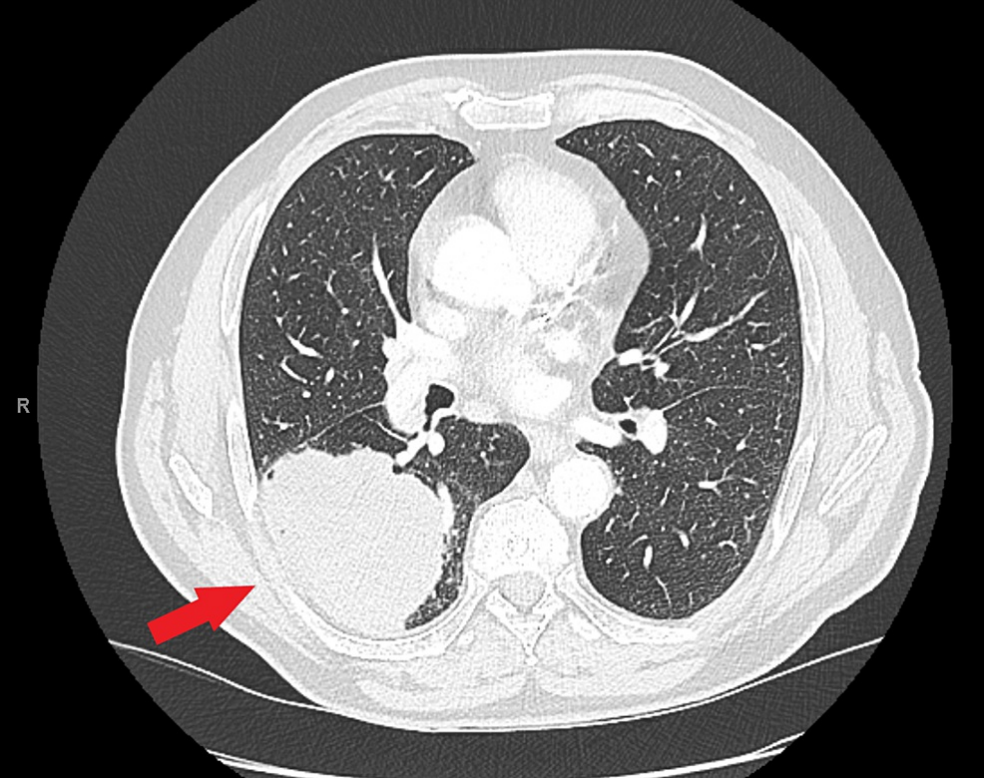
Chest CT scan Chest CT demonstrating a 9-centimeter pulmonary mass located in the right lower lobe (red arrow)

**Figure 3:**
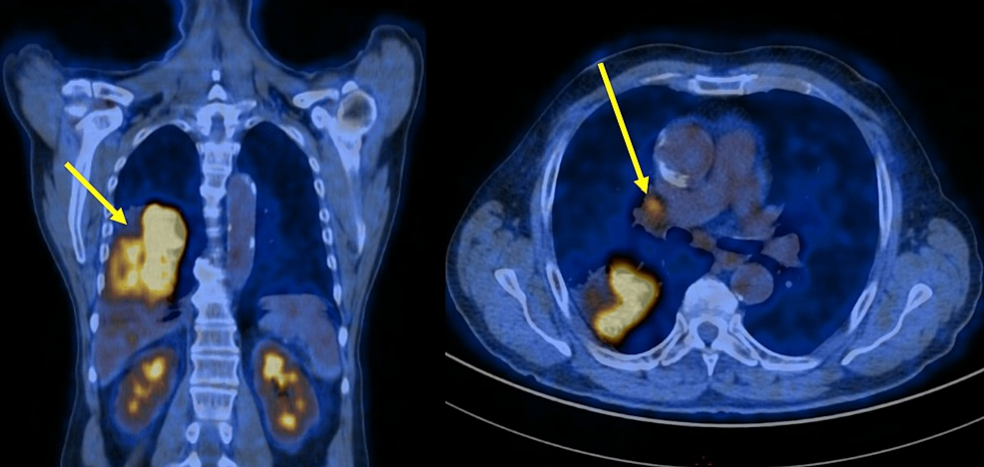
Preoperative PET-CT scan Demonstration of high fluorodeoxyglucose (FDG) uptake in the mass of the right lower lobe (left arrow) associated with ipsilateral hilar adenopathy (right arrow)

A chest CT showed a 9-centimeter pulmonary mass located in the right lower lobe (Figure 2). A PET-CT confirmed a strong 18F-fluorodésoxyglucose uptake of the lesion as well as an ipsilateral hypermetabolic hilar lymphadenopathy without signs of mediastinal adenopathy or distant metastases (Figures 2, 3). Brain magnetic resonance imaging (MRI) was negative for metastatic lesions. Endobronchial ultrasound-guided (EBUS) transbronchial biopsies were performed and confirmed the presence of an adenocarcinoma in the right lower lobe. Hilar and mediastinal lymph nodes were assessed and were found to be metastasis-free.

**Figure 4:**
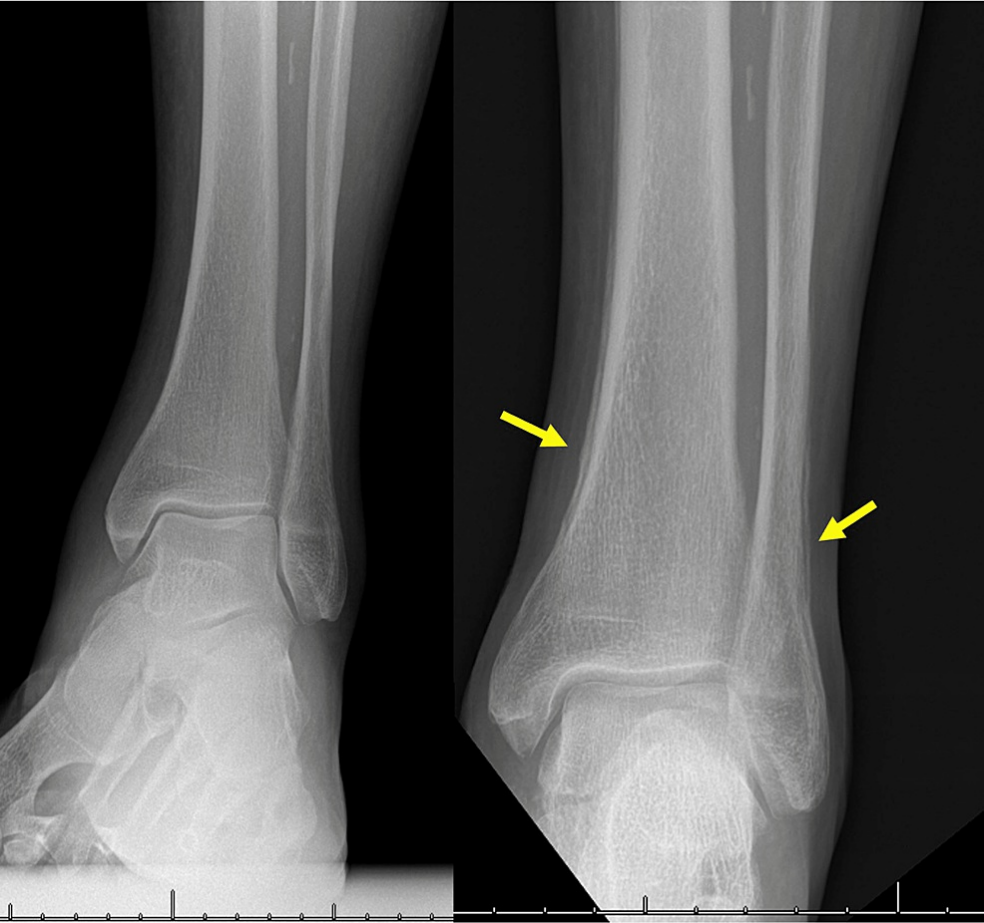
Radiological osteoarticular manifestations of the Pierre-Marie Bamberger syndrome X-ray of the left ankle in five-month intervals demonstrating the appearance of hypertrophic osteoarthropathy (arrows) and periostosis of the tibia and fibula

Further investigations included a lower limb bilateral X-ray showing osteoarthropathy of the distal tibia not present on X-rays carried out five months earlier (Figure 4). Bone scintigraphy was also carried out and returned normal. After cardiopulmonary evaluation and discussion during our institution’s multidisciplinary oncological board, upfront surgery with right lower lobectomy associated with mediastinal lymph node dissection was performed through a right posterolateral thoracotomy. The patient had an uneventful course and was discharged on postoperative day 6. Histological assessment of the surgical specimen revealed a mixed pulmonary adenocarcinoma (40% papillary, 30% micropapillary, and 30% acinar) of 9 cm classified as pT4 pN2 (16/30) L0 V0 Pn0 PL0 with complete resection R0 (stage IIIB according to 8th TNM edition); node harvesting included two positive intraparenchymal lymph nodes (station 12) and one peri-hilar lymph node (Station 10) (3/3), one interlobar lymph node (station 11) (1/1), seven hilar lymph nodes (Station 10) (7/7), five carinal lymph nodes (Station 7) (5/12) and zero lower and upper paratracheal lymph nodes (Station 2R and 4R) (0/7). Adjuvant chemotherapy treatment was delivered. During the postoperative follow-up at one month, the patient showed remission of HOA marked with the complete disappearance of digital clubbing and osteoarticular symptoms. The patient died six months after surgery due to a stroke during hospitalization for moderate acute respiratory distress syndrome (ARDS) in the context of pneumonia.

## Discussion

A fully developed HOA in the context of Pierre-Marie Bamberger syndrome is a rare finding and appears in approximately 0.8% of patients with lung cancer. However, when actively researched, signs and symptoms of Pierre-Marie Bamberger syndrome may be present in up to 5% of cases of lung cancer [4,7,8]. Besides lung cancer, the syndrome has been reported in the presence of other malignancies such as mesothelioma and thymoma [2-7].

Several reports of this rare syndrome mention articular pain as the initial and main symptom, subsequently leading to the diagnosis of an underlying lung neoplasia [10-13]. Similarly, in our patient’s case, articular pain and edema were the symptoms that prompted our patient to consult his general practitioner, leading to further investigations and the discovery of a pulmonary neoplasm. Hemoptysis was only discovered during medical history and was not the main motive of consultation. Clubbing of fingers associated with synovial effusion and periostosis should raise suspicion of a so far undiagnosed lung malignancy.

First described by Hippocrates over 2500 years ago, digital clubbing is considered one of the oldest signs in clinical medicine. However, the physiopathology of digital clubbing still remains unknown and the same applies to the rest of the findings associated with HOA. Several underlying mechanisms associated with HOA development have been investigated, including abnormal vascularization, hypoxia, and chronic inflammation [2,7,14-16]. Hypoxia constitutes the main pathophysiological mechanism leading to the development of HOA in the setting of chronic obstructive pulmonary disease, cystic fibrosis, and congenital cyanotic heart disease. On the other hand, the syndrome has been described in association with inflammatory bowel disease and polyarteritis nodosa where chronic inflammation is believed to be the main pathophysiological pattern rather than hypoxia [6]. Concerning lung cancer, both hypoxia and chronic inflammation contribute to the appearance of HOA [7].

Other pathophysiological pathways have also been explored, such as tumor-related thrombocyte and megakaryocyte activation, releasing, in turn, different growth factors. The most commonly involved growth factors seem to be the platelet-derived growth factor (PDGF) and vascular endothelial growth factor (VEGF), both of which promote mesenchymal cell growth [2,7]. Systemic prostaglandin E2 (PGE2) levels have also been found to be markedly increased in patients with HOA in the context of lung cancer. This appears to be related to the presence of cyclooxygenase-2 (Cox-2), which is expressed by the tumor and is involved in the PGE2 synthesis [15].

In fact, Cox-2 inhibitors are known to rapidly decrease HOA symptoms [16]. The same applies in the case of Cox-2-producing tumors, where surgical removal results in quick and permanent disappearance of HOA symptoms [7,15,17]. As tumor overexpression of Cox-2 has been associated with poor outcomes in NSCLC patients, Cox-2 inhibitors have been the focus of different immunotherapy and chemotherapy agents. Unfortunately, clinical trials aiming at Cox-2 inhibitor pathways have not shown to be successful in improving survival in patients with NSCLC [18-20]. In our case, Cox-2 research on the surgical specimen could not be carried out.

The discovery of this syndrome associated with our patient’s lung neoplasm mainly lies in the initial clinical appearance associated with the complete remission of osteoarticular signs and symptoms following the complete surgical removal of the tumor. The attenuation of signs and symptoms of the Pierre-Marie Bamberger syndrome following treatment of the underlying pathology has been widely reported in the current literature [10-13]. On the other hand, symptoms persist and seem to progress in cases of patients receiving palliation treatment, where the underlying cause remains untreated [2,11-13].

Finally, the literature reports that the signs and symptoms of HOA do not recur even in the case of subsequent metastatic disease as long as there is no Cox-2 expression by the metastatic lesions [17]. In this case report, the patient presented a complete Pierre-Marie Bamberger syndrome related to a localized NSCLC with complete remission of signs and symptoms following the surgical resection of the underlying tumor.

## Conclusions

Awareness of Pierre-Marie Bamberger syndrome may help detect underlying lung malignancies. When possible, histological and molecular examination of the surgical specimen should include Cox-2 analysis in order to confirm the diagnosis of HOA and help further elucidate the Cox-2 pathway in the development of the syndrome in the context of lung diseases. This report has highlighted the importance of clinical awareness of the association between HOA and carcinoma of the lung.
